# Efficacy of Delafloxacin versus Moxifloxacin against Bacterial Respiratory Pathogens in Adults with Community-Acquired Bacterial Pneumonia (CABP): Microbiology Results from the Delafloxacin Phase 3 CABP Trial

**DOI:** 10.1128/AAC.01949-19

**Published:** 2020-02-21

**Authors:** Sandra McCurdy, Kara Keedy, Laura Lawrence, Ashley Nenninger, Amanda Sheets, Megan Quintas, Sue Cammarata

**Affiliations:** a Melinta Therapeutics, Morristown, New Jersey, USA

**Keywords:** community-acquired bacterial pneumonia, clinical trial, fluoroquinolone, delafloxacin

## Abstract

Delafloxacin is a novel fluoroquinolone with activity against Gram-positive, Gram-negative, and atypical pathogens, including fluoroquinolone-nonsusceptible methicillin-resistant Staphylococcus aureus (MRSA). The microbiological results of a phase 3 clinical trial in adults with community-acquired pneumonia (CAP) comparing delafloxacin (300 mg intravenously [i.v.] with the option to switch to 450 mg orally every 12 h) to moxifloxacin (400 mg i.v. with the option to switch to 400 mg orally once a day [QD]) were determined. Patients from 4 continents, predominately Europe but also South America and Asia, were enrolled. The microbiological intent-to-treat (MITT) population included 520 patients, and 60.5% of these patients had a bacterial pathogen identified. Multiple diagnostic methods were employed, including culture, serology, PCR, and urinary antigen tests. Based on baseline MIC_90_ values, delafloxacin exhibited at least 16-fold greater activity than moxifloxacin for Gram-positive and fastidious Gram-negative pathogens. Delafloxacin retained activity against resistant phenotypes found in Streptococcus pneumoniae (penicillin-, macrolide-, and multiple-drug resistant), *Haemophilus* species (β-lactamase producing and macrolide nonsusceptible), and S. aureus (MRSA and fluoroquinolone-nonsusceptible methicillin-susceptible S. aureus [MSSA]). The microbiological success rates were 92.7% for S. pneumoniae (87.5% for penicillin-resistant S. pneumoniae [PRSP]), 92.6% for S. aureus (100% for MRSA), 100% for Escherichia coli, 82.4% for Klebsiella pneumoniae, 100% for Klebsiella oxytoca, 100% for Moraxella catarrhalis, 91.7% for Haemophilus influenzae, 88.6% for Haemophilus parainfluenzae, 96.7% for Mycoplasma pneumoniae, 93.1% for Legionella pneumophila, and 100% for Chlamydia pneumoniae. There was little correlation between MICs and outcomes, with a high proportion of favorable outcomes observed across all delafloxacin baseline MIC values. Delafloxacin may be considered a treatment option as monotherapy for CAP in adults, where broad-spectrum coverage including MRSA activity is desirable.

## Abstract (precis)

Delafloxacin is a novel fluoroquinolone with activity against Gram-positive, Gram-negative, and atypical pathogens, including fluoroquinolone-nonsusceptible methicillin-resistant Staphylococcus aureus (MRSA). The microbiological results of a phase 3 clinical trial in adults with community-acquired pneumonia (CAP) comparing delafloxacin (300 mg intravenously [i.v.] with the option to switch to 450 mg orally every 12 h) to moxifloxacin (400 mg i.v.

## INTRODUCTION

Each year, 5 million to 10 million patients are treated for community-acquired pneumonia (CAP) in the United States (1), with medical costs exceeding $10 billion in 2011 (2). According to the Centers for Disease Control and Prevention (CDC), there were 49,157 deaths and 257,000 visits to the emergency department with pneumonia as the primary diagnosis (https://www.cdc.gov/nchs/fastats/pneumonia.htm).

In the Etiology of Pneumonia in the Community (EPIC) study, certain pathogens were isolated with increased frequencies in intensive care unit (ICU) patients over non-ICU patients: Streptococcus pneumoniae (8% versus 4%), Staphylococcus aureus (5% versus 1%), and *Enterobacteriaceae* (3% versus 1%) (3). A follow-up analysis of the EPIC data revealed that among 2,259 adults hospitalized for community-acquired pneumonia, 37 (1.6%) had S. aureus identified, including 15 (0.7%) with methicillin-resistant S. aureus (MRSA) and 22 (1.0%) with methicillin-susceptible S. aureus (MSSA), while 115 (5.1%) had S. pneumoniae identified. In addition, patients with CAP caused by S. aureus, especially MRSA, tended to have higher severity scores than patients with non-S. aureus and pneumococcal CAP (4). The EPIC study demonstrated that while MRSA is an infrequent pulmonary pathogen, it is an important pathogen to detect because it can be associated with severe disease and may be resistant to standard antibiotics for CAP.

Delafloxacin is a novel fluoroquinolone antibiotic that possesses activity against Gram-positive, Gram-negative, and atypical pathogens, including activity against fluoroquinolone-nonsusceptible MRSA isolates (5). It is FDA approved for the treatment of patients with acute bacterial skin and skin structure infections (ABSSSIs) and community-acquired bacterial pneumonia (CABP) (6). In a recently completed phase 3 CABP study, delafloxacin was noninferior to moxifloxacin for the primary endpoint, the early clinical response (ECR) (88.9 versus 89.0; 95% confidence interval [CI], −4.4, 4.1). A detailed analysis of the microbiology results from the phase 3 study (ML-3341-306; ClinicalTrials.gov identifier NCT02679573) was conducted and is presented here.

## RESULTS

### Diagnostic yield.

Of 859 patients in the intent-to-treat (ITT) population, 520 patients (60.5%) had at least 1 pathogen detected at baseline by any method (including respiratory or blood culture, PCR, serology, and urinary antigen tests [UATs]) and thus comprised the microbiological intent-to-treat subgroup 1 (MITT-1) population. A total of 90.2% (469/520) of the MITT-1 population had a definitive pathogen detected. Table 1 presents comparisons of the yields for these pathogens by the diagnostic methods employed in the trial and by definitive diagnoses in the MITT-1 population. In the delafloxacin treatment group, for S. pneumoniae, the nasopharyngeal (NP) swab culture/PCR methodology yielded the highest number of S. pneumoniae diagnoses (21.8%), followed by urinary antigen testing (17.1%) and sputum culture (11.7%). For Mycoplasma pneumoniae (12.5%) and Legionella pneumophila (10.1%), serology yielded the highest number of diagnoses. Diagnostic yields were generally comparable between treatment groups, except for S. pneumoniae diagnosis by urinary antigen testing, where the delafloxacin treatment group had slightly more diagnoses using this method than the moxifloxacin arm (17.1% versus 11.8%).

**TABLE 1:** Comparison of diagnostic method yields by baseline pathogen and by analysis population (MITT-1)^*a*^

Pathogen and method of isolation	No. (%) of MITT-1 patients			
	Delafloxacin (*n* = 257)		Moxifloxacin (*n* = 263)	
	All diagnoses	Definitive diagnoses	All diagnoses	Definitive diagnoses
S. pneumoniae	120 (46.7)	98 (38.1)	106 (40.3)	83 (31.6)
Sputum culture	30 (11.7)	25 (9.7)	37 (14.1)	33 (12.5)
Blood culture	4 (1.6)	4 (1.6)	6 (2.3)	6 (2.3)
Bronchoalveolar lavage fluid culture	3 (1.2)	3 (1.2)	0	0
NP swab culture with *lytA* PCR (>1,000 gene copies/ml)	56 (21.8)	56 (21.8)	47 (17.9)	47 (17.9)
NP swab *lytA* PCR	17 (6.6)		20 (7.6)	
Urinary antigen test	44 (17.1)	44 (17.1)	31 (11.8)	31 (11.8)

Legionella pneumophila	29 (11.3)	29 (11.3)	33 (12.5)	33 (12.5)
Sputum culture	4 (1.6)	4 (1.6)	1 (0.4)	1 (0.4)
Urinary antigen test	8 (3.1)	8 (3.1)	6 (2.3)	6 (2.3)
Serology	26 (10.1)	26 (10.1)	31 (11.8)	31 (11.8)

Mycoplasma pneumoniae	35 (13.6)	35 (13.6)	30 (11.4)	30 (11.4)
Oropharyngeal swab culture	11 (4.3)	11 (4.3)	12 (4.6)	12 (4.6)
Serology	32 (12.5)	32 (12.5)	27 (10.3)	27 (10.3)

Chlamydia pneumoniae	25 (9.7)	25 (9.7)	16 (6.1)	16 (6.1)
Serology	25 (9.7)	25 (9.7)	16 (6.1)	16 (6.1)

a Patients with the same pathogen detected by multiple methods are counted once in the overall category and once for each diagnostic method with a positive result. A pathogen is considered “definitive” if at least one microbiological diagnosis is “definitive”; a pathogen is considered “probable” if all microbiological diagnoses are “probable.” Small numbers of patients (<10%) had probable diagnoses and thus are not included in the table. NP, nasopharyngeal.

### Monomicrobial versus polymicrobial infections.

The presence of monomicrobial Gram-positive, Gram-negative, and atypical infections and polymicrobial infections was as follows. In the MITT-1 (definitive diagnoses) and microbiologically evaluable test-of-cure subgroup 2 (ME-2TOC) populations in the delafloxacin arm, 26.4% (61/231) and 33.7% (64/190) of patients had monomicrobial Gram-positive infections, respectively. In the MITT-1 (definitive diagnoses) and ME-2TOC populations in the delafloxacin arm, 20.3% (47/231) and 38.4% (73/190) had monomicrobial Gram-negative infections, 13.4% (31/231) and 4.7% (9/190) had atypical infections, and 39.8% (92/231) and 23.2% (44/190) had polymicrobial infections. There were no major differences in the percentages of patients with monomicrobial and polymicrobial infections between the two treatment arms.

### Antimicrobial susceptibility results.

The *in vitro* activity of delafloxacin against baseline pathogens is shown in Table 2. Based on MIC_90_ values at baseline, delafloxacin exhibited at least 16-fold greater activity than moxifloxacin for all Gram-positive and fastidious Gram-negative pathogens in the MITT-2 population (see the supplemental material). Most of these isolates were fluoroquinolone susceptible, macrolide susceptible, and penicillin susceptible (penicillin-susceptible S. pneumoniae [PSSP]); methicillin susceptible (MSSA); or β-lactamase negative (*Haemophilus* spp.). However, delafloxacin retained activity against resistant phenotypes found in S. pneumoniae (penicillin-resistant S. pneumoniae [PRSP], penicillin-intermediate S. pneumoniae [PISP], macrolide-nonsusceptible S. pneumoniae, and multidrug-resistant S. pneumoniae), *Haemophilus* species (β-lactamase positive and macrolide nonsusceptible), S. aureus (MRSA and fluoroquinolone-nonsusceptible MSSA), and M. pneumoniae (macrolide resistant) (Table 2).

**TABLE 2:** Delafloxacin MICs against baseline pathogens (MITT-2 [moxifloxacin and delafloxacin treatment groups combined])^*a*^

Baseline target pathogen	No. of infections	Delafloxacin MIC range (μg/ml)	Delafloxacin MIC_50_ (μg/ml)^*b*^	Delafloxacin MIC_90_ (μg/ml)^*b*^
Gram-positive organisms				
Streptococcus pneumoniae	142	0.004 to 0.03	0.015	0.015
PSSP	102	0.004 to 0.03	0.015	0.015
PISP	25	0.008 to 0.03	0.015	0.015
PRSP	19	0.004 to 0.015	0.015	0.015
MDR	12	0.004 to 0.015	0.015	0.015
Macrolide susceptible	108	0.004 to 0.03	0.015	0.015
Macrolide nonsusceptible	34	0.004 to 0.015	0.015	0.015
Staphylococcus aureus	57	0.001 to 0.12	0.002	0.004
MSSA	55	0.001 to 0.12	0.002	0.004
MRSA	2	0.002 to 0.004	—	—
Fluoroquinolone susceptible	54	0.001 to 0.008	0.002	0.004
Fluoroquinolone nonsusceptible	3	0.12 to 0.12	—	—

Gram-negative fastidious organisms				
Haemophilus parainfluenzae	75	0.0005 to 4	0.008	0.5
Macrolide susceptible	67	0.0005 to 4	0.008	0.5
Macrolide nonsusceptible	8	0.002 to 2	—	—
β-Lactamase negative	71	0.0005 to 4	0.008	0.5
β-Lactamase positive	4	0.001 to 0.008	—	—
Haemophilus influenzae	61	0.00025 to 0.5	0.001	0.002
Macrolide susceptible	59	0.00025 to 0.5	0.0005	0.002
Macrolide nonsusceptible	2	0.001 to 0.002	—	—
β-Lactamase negative	58	0.00025 to 0.5	0.0005	0.002
β-Lactamase positive	3	0.0005 to 0.002	—	—
Moraxella catarrhalis	12	0.002 to 0.015	0.004	0.004

Gram-negative organisms				
Klebsiella pneumoniae	33	0.03 to >256	0.12	0.25
Fluoroquinolone susceptible	31	0.03 to 2	0.12	0.25
Fluoroquinolone nonsusceptible	2	>256 to >256	—	—
ESBL negative	29	0.03 to 2	0.12	0.25
ESBL positive	4	0.12 to >256	—	—
Escherichia coli	27	0.015 to 4	0.06	4
Fluoroquinolone susceptible	24	0.015 to 4	0.03	0.06
Fluoroquinolone nonsusceptible	3	4 to 4	—	—
ESBL negative	23	0.03 to 4	0.06	0.06
ESBL positive	4	0.015 to 4	—	—
Enterobacter cloacae complex	14	0.06 to 256	0.12	0.25
Fluoroquinolone susceptible	13	0.06 to 0.25	0.12	0.25
Fluoroquinolone nonsusceptible	1	256	—	—
Klebsiella oxytoca	10	0.06 to 4	0.12	2
Pseudomonas aeruginosa	24	0.008 to 16	0.5	4
Fluoroquinolone susceptible	19	0.008 to 4	0.25	2
Fluoroquinolone nonsusceptible	5	1 to 16	—	—

Atypical organisms				
Mycoplasma pneumoniae	19	0.125 to 0.5	0.25	0.5
Macrolide susceptible	17	0.125 to 0.5	0.25	0.5
Macrolide resistant	2	0.125 to 0.25	—	—
Legionella pneumophila	5	0.00025 to 0.001	—	—

a Abbreviations: —, not applicable; MDR, multiple-drug resistant; MRSA, methicillin-resistant S. aureus; MSSA, methicillin-susceptible S. aureus; PISP, penicillin-intermediate S. pneumoniae; PRSP, penicillin-resistant S. pneumoniae; PSSP, penicillin-susceptible S. pneumoniae.

b MIC_50_ and MIC_90_ values were calculated only when 10 or more isolates were available.

### Efficacy analysis for delafloxacin.

The analysis of the per-pathogen microbiological response observed for delafloxacin- and moxifloxacin-treated patients at baseline is presented in Table 3. Overall, there was a high degree of favorable response at the test of cure (TOC) for delafloxacin-treated patients. By pathogen, the rates of microbiological success (documented or presumed eradication) at TOC were similar between the delafloxacin group and the moxifloxacin group for the most common pathogens (occurring in >5 patients in either treatment group) in the ME-1TOC population (Table 3). Similar findings were observed in the MITT-1, MITT-2, and ME-2TOC populations (data not shown) and in those patients who had a definitive diagnosis versus patients who had either a probable or definitive diagnosis (all diagnoses). Approximately one-third of patients in the delafloxacin (83/240; ME-1TOC) and moxifloxacin (76/248; ME-1TOC) treatment groups had atypical pathogens, and high rates of response were also observed for these pathogens.

**TABLE 3:** Per-pathogen microbiological response at the test of cure by the most common baseline pathogens (ME-1TOC [delafloxacin and moxifloxacin treatment groups])^*a*^

Pathogen	No. of ME-1TOC patients with success/total no. of ME-1TOC patients (%)^*b*^			
	All diagnoses		Definitive diagnoses	
	Delafloxacin (*n* = 240)	Moxifloxacin (*n* = 248)	Delafloxacin (*n* = 240)	Moxifloxacin (*n* = 248)
Gram-positive organisms				
Streptococcus pneumoniae^*c*^	102/110 (92.7)	93/99 (93.9)	85/91 (93.4)	72/77 (93.5)
PSSP	46/49 (93.9)	44/47 (93.6)	45/47 (95.7)	41/44 (93.2)
PISP	16/17 (94.1)	6/7 (85.7)	13/14 (92.9)	5/6 (83.3)
PRSP	7/8 (87.5)	11/11 (100.0)	7/8 (87.5)	11/11 (100.0)
MDRSP	4/4 (100.0)	8/8 (100.0)	4/4 (100.0)	8/8 (100.0)
Macrolide resistant	15/17 (88.2)	17/18 (94.4)	15/16 (93.8)	17/18 (94.4)
Culture diagnosis (ME-2TOC)	66/71 (93.0)	60/64 (93.8)	66/71 (93.0)	60/64 (93.8)
Staphylococcus aureus^*c*^	25/27 (92.6)	28/30 (93.3)	23/25 (92.0)	23/25 (92.0)
MSSA	23/25 (92.0)	28/30 (93.3)	21/23 (91.3)	23/25 (92.0)
MRSA	2/2 (100.0)	0	2/2 (100.0)	0

Gram-negative fastidious organisms				
Haemophilus parainfluenzae	31/35 (88.6)	32/37 (86.5)	27/31 (87.1)	30/34 (88.2)
Haemophilus influenzae	22/24 (91.7)	31/35 (88.6)	17/19 (89.5)	27/30 (90.0)
Moraxella catarrhalis	6/6 (100.0)	6/6 (100.0)	4/4 (100.0)	5/5 (100.0)

Gram-negative organisms				
Klebsiella pneumoniae	14/17 (82.4)	16/16 (100.0)	13/15 (86.7)	16/16 (100.0)
Escherichia coli	13/13 (100.0)	9/9 (100.0)	13/13 (100.0)	6/6 (100.0)
Pseudomonas aeruginosa	11/12 (91.7)	11/11 (100.0)	9/10 (90.0)	11/11 (100.0)
Klebsiella oxytoca	6/6 (100.0)	3/4 (75.0)	6/6 (100.0)	3/4 (75.0)
Enterobacter cloacae complex	3/4 (75.0)	8/8 (100.0)	2/3 (66.7)	7/7 (100.0)

Atypical organisms				
Mycoplasma pneumoniae	29/30 (96.7)	29/29 (100.0)	29/30 (96.7)	29/29 (100.0)
Culture diagnosis (ME-2TOC)	11/11 (100.0)	11/11 (100.0)	11/11 (100.0)	11/11 (100.0)
Legionella pneumophila	27/29 (93.1)	32/32 (100.0)	27/29 (93.1)	32/32 (100.0)
Chlamydia pneumoniae	24/24 (100.0)	15/15 (100.0)	24/24 (100.0)	15/15 (100.0)

a Abbreviations: ME, microbiologically evaluable; TOC, test of cure; PSSP, penicillin-susceptible S. pneumoniae; PISP, penicillin-intermediate S. pneumoniae; PRSP, penicillin-resistant S. pneumoniae; MDRSP, multiple-drug-resistant S. pneumoniae; MRSA, methicillin-resistant S. aureus; MSSA, methicillin-susceptible S. aureus.

b Success was defined as documented or presumed eradication.

c Patients with any combination of PSSP, PISP, or PRSP were counted once in the overall category for that organism.

### Efficacy by monomicrobial or polymicrobial infection.

Microbiological responses (ME-1TOC) by baseline monomicrobial and polymicrobial infection for patients with a definitive diagnosis were similar between delafloxacin- and moxifloxacin-treated subjects (Table 4). Among monomicrobial infections, response rates were high for pathogens in the delafloxacin treatment group, at >90% for S. pneumoniae, Escherichia coli, Pseudomonas aeruginosa, Klebsiella oxytoca, Moraxella catarrhalis, and the atypical pathogens M. pneumoniae, L. pneumophila, and Chlamydia pneumoniae. Per-pathogen response rates were similarly high for polymicrobial infections, at >90% for S. pneumoniae, S. aureus (MSSA and MRSA), Haemophilus influenzae, E. coli, K. oxytoca, M. catarrhalis, the Enterobacter cloacae complex, and the atypical pathogens M. pneumoniae, L. pneumophila, and C. pneumoniae.

**TABLE 4:** Microbiological responses at the test of cure by baseline monomicrobial versus polymicrobial infections (ME1-TOC, definitive diagnosis [delafloxacin and moxifloxacin treatment groups])

Type of infection	No. of patients with definitive diagnosis with microbiological response/total no. of patients (%)	
	Delafloxacin (*n* = 240)	Moxifloxacin (*n* = 248)
Monomicrobial		
Gram positive	55/58 (94.8)	50/55 (90.9)
Gram negative	39/45 (86.7)	51/56 (91.1)
Atypical	29/30 (96.7)	35/35 (100)

Polymicrobial	80/86 (93.0)	75/79 (94.9)

### Outcomes by MIC.

The analysis of the per-pathogen microbiological response by baseline MIC value observed for delafloxacin-treated patients with definitive pathogen diagnoses in the ME-2TOC population is shown in Table 5. By analyzing the microbiological outcome data using definitive diagnoses only, the most conservative analysis of the microbiological outcome data is presented for each species. Given the high overall rates of success observed, there was little correlation observed between MIC and outcome, with a high proportion of favorable outcomes being observed across all delafloxacin MIC values at baseline. Favorable eradication rates were also observed for S. pneumoniae isolates with resistant phenotypes, including PRSP (87.5% [7/8]), multiple-drug-resistant S. pneumoniae (MDRSP) (100.0% [4/4]), and macrolide-resistant S. pneumoniae (MRSP) (88.2% [15/17]), with no apparent correlation between outcome and MIC value. No fluoroquinolone-nonsusceptible S. pneumoniae isolates were recovered. Most of the S. aureus isolates were MSSA (23/25), and only 2 isolates were MRSA. Both MRSA isolates had an outcome of eradication/presumed eradication with delafloxacin MIC values of 0.002 μg/ml and 0.004 μg/ml. One of the MSSA isolates was fluoroquinolone resistant (delafloxacin MIC = 0.12 μg/ml; moxifloxacin MIC = 2 μg/ml; levofloxacin MIC = 4 μg/ml). This isolate was found to have quinolone resistance-determining region (QRDR) mutations in *grlA* (S80F), *grlB* (D422E), and *gyrA* (S84L) by whole-genome sequencing (WGS). Despite these QRDR mutations, the microbiological outcome was eradicated/presumed eradicated for this isolate. This finding corroborates previously observed data for S. aureus isolates in the delafloxacin ABSSSI trial (5). For H. influenzae, favorable outcomes were prevalent across all MIC values except for a single isolate with an MIC value of 0.5 μg/ml. This isolate was moxifloxacin and levofloxacin nonsusceptible and was found to have QRDR mutations in *gyrA* (S84L and D88G), *parC* (S84I), and *parE* (D420N) by WGS. For Haemophilus parainfluenzae, favorable outcomes were prevalent across most MIC values except for isolates with delafloxacin MIC values of 0.015 μg/ml and 0.25 μg/ml. By WGS, the isolate with an MIC of 0.015 μg/ml was found to be a wild-type isolate, while the isolate with an MIC of 0.25 μg/ml had a QRDR mutation in *gyrA* (S84F). In the delafloxacin treatment group, one isolate developed resistance upon therapy (>4-fold increase in the MIC). The isolate recovered during the end-of-therapy (EOT) visit had a delafloxacin MIC that was 64-fold higher than that of the baseline isolate (baseline delafloxacin MIC = 0.015 μg/ml; EOT delafloxacin MIC = 1 μg/ml). Both isolates were screened for fluoroquinolone resistance mechanisms by WGS analysis. While the baseline isolate was confirmed to be a wild-type isolate, the isolate recovered at EOT had mutations in the *gyrA* (S84F and D88Y) and *parC* (S84Y) genes. Both the baseline isolate and the persistent isolate were subjected to pulsed-field gel electrophoresis (PFGE), and the isolates were found to be genetically related. A >4-fold increase in fluoroquinolone MIC values was observed in 4 H. parainfluenzae isolates from moxifloxacin-treated patients; however, the paired isolates were found to be unrelated by PFGE. No other organisms showed a >4-fold increase in MIC values in this study.

**TABLE 5:** Correlation of delafloxacin baseline MICs with microbiological eradication rates at TOC (ME-2TOC, definitive diagnosis [delafloxacin treatment group]) for proposed CABP indication pathogens^*a*^

Organism	No. of patients demonstrating microbiological eradication/total no. of patients (%) at MIC (μg/ml) of:																
	All	0.00025	0.0005	0.001	0.002	0.004	0.008	0.015	0.03	0.06	0.12	0.25	0.5	1	2	4	>4
Gram-positive organisms																	
S. pneumoniae	62/66 (93.9)					2/2 (100)	19/20 (95.0)	39/41 (95.1)	2/3 (66.7)								
S. aureus	23/25 (92.0)			5/5 (100)	8/10 (80.0)	9/9 (100)					1/1 (100)						
MRSA	2/2 (100)				1/1 (100)	1/1 (100)											
MSSA	21/23 (91.3)			5/5 (100)	7/9 (77.8)	8/8 (100)					1/1 (100)						

Gram-negative organisms																	
K. pneumoniae	13/15 (86.7)									4/4 (100)	6/8 (75.0)	1/1 (100)			1/1 (100)		1/1 (100)
K. oxytoca	6/6 (100)									3/3 (100)	1/1 (100)	1/1 (100)				1/1 (100)	
E. coli	13/13 (100)								5/5 (100)	5/5 (100)	1/1 (100)					2/2 (100)	
P. aeruginosa	9/10 (90)						1/1 (100)				1/1 (100)	3/3 (100)	1/2 (50)	1/1 (100)	1/1 (100)	1/1 (100)	

Gram-negative organisms (fastidious)																	
H. influenzae	17/19 (89.5)	1/1 (100)	6/6 (100)	9/10 (90.0)	1/1 (100)								0/1 (0)				
*H. parainfluenzae*	26/30 (86.7)		1/1 (100)	3/3 (100)	4/5 (80.0)	6/6 (100)	7/8 (87.5)	2/3 (66.7)		1/1 (100)		0/1 (0)	1/1 (100)			1/1 (100)	
M. catarrhalis	4/4 (100)				2/2 (100)	2/2 (100)											

Atypical organisms																	
M. pneumoniae	7/7 (100)										1/1 (100)	4/4 (100)	2/2 (100)				
L. pneumophila	3/4 (75.0)	1/1 (100)	1/2 (50)	1/1 (100)													

a Abbreviations: MRSA, methicillin-resistant S. aureus; MSSA, methicillin-susceptible S. aureus.

## DISCUSSION

These data demonstrated the overall efficacy of intravenous (i.v.)/oral delafloxacin monotherapy in the treatment of patients with CABP. Delafloxacin was noninferior to moxifloxacin in the primary endpoint, the early clinical response. Based on MIC_90_ values at baseline, delafloxacin exhibited at least 16-fold greater activity than moxifloxacin for all Gram-positive and fastidious Gram-negative pathogens in the culture-based MITT-2 population (see the supplemental material). Delafloxacin and moxifloxacin had similar activities against M. pneumoniae isolates (including 2 macrolide-resistant isolates), and delafloxacin had greater activity than moxifloxacin against L. pneumophila isolates (see the supplemental material). Delafloxacin retained activity against resistant phenotypes found in S. pneumoniae (penicillin resistant, macrolide resistant, and multiple-drug resistant), *Haemophilus* species (β-lactamase producing and macrolide nonsusceptible), and S. aureus (MRSA and fluoroquinolone-nonsusceptible MSSA). As noted above, no fluoroquinolone-nonsusceptible S. pneumoniae isolates were recovered from this CABP clinical trial. This finding is not unusual, as fluoroquinolone-nonsusceptible S. pneumoniae isolates were also not recovered from patients in the LEAP 1 (7) and the OPTIC (8) CABP clinical trials. Overall, among 142 baseline S. pneumoniae isolates with susceptibility test results available, resistance rates were 24.6% for macrolide resistance, 13.4% for penicillin resistance, and 8.5% for multidrug resistance. The MRSA and fluoroquinolone-nonsusceptible MSSA MIC and QRDR data corroborated previous findings from the ABSSSI clinical trial, where delafloxacin demonstrated high rates of microbiological response against levofloxacin-nonsusceptible isolates as well as isolates with documented mutations in the QRDR. Most of the *Enterobacteriaceae* and P. aeruginosa isolates were fluoroquinolone susceptible. Delafloxacin demonstrated reduced activity against some isolates with fluoroquinolone-nonsusceptible and extended-spectrum-β-lactamase (ESBL) phenotypes (Table 2).

By pathogen, the rates of microbiological success (documented or presumed eradicated) at TOC were similar between the delafloxacin group and the moxifloxacin group for the most common pathogens in the MITT-1, MITT-2, ME-1TOC, and ME-2TOC populations. For the ME-1TOC population, the microbiological success rates were 92.7% for S. pneumoniae (87.5% for PRSP), 92.6% for S. aureus (100% for MRSA), 100% for E. coli, 82.4% for K. pneumoniae, 100% for K. oxytoca, 100% for M. catarrhalis, 91.7% for H. influenzae, and 88.6% for *H. parainfluenzae*. For the atypical pathogens, the microbiological success rates were 96.7% for M. pneumoniae, 93.1% for L. pneumophila, and 100% for C. pneumoniae. There was little correlation observed between MICs and outcomes, with a high proportion of favorable outcomes observed across all delafloxacin MIC values at baseline.

For the *H. parainfluenzae* isolate in the delafloxacin arm where emergence of resistance was observed, it is noteworthy that the patient was successfully treated, and the clinical response at both EOT and TOC was success. Interestingly, for the isolates with a >4-fold increase in fluoroquinolone MIC values from both arms, 3 out of the 4 isolates were genetically unrelated. Previous studies from Spain and South Africa reported fluoroquinolone resistance arising from mutations in the QRDR as well as plasmid-mediated resistance (9, 10). The mutations observed in *H. parainfluenzae* in this study were also observed in isolates reported in the Spanish and South African studies. Since the pneumonia was community acquired, this could explain the nonclonal nature of the isolates from the moxifloxacin arm.

In conclusion, these data suggest that delafloxacin may be considered a treatment option as monotherapy for CAP in adults, where broad-spectrum coverage is desirable.

## MATERIALS AND METHODS

### Study design and efficacy endpoints.

Delafloxacin was studied in patients with CABP in one phase 3, multicenter, stratified, randomized, double-blind trial designed using the guidelines of the FDA (11) and the European Medicines Agency (12). A total of 859 adult patients from sites on 4 continents, including sites in the United States (0.7%), Europe (85.7%), Latin America (5.4%), and South Africa (8.3%), were enrolled. The enrollment period spanned from December 2016 to July 2018. Patients with CABP were randomly assigned in a 1:1 ratio to receive either delafloxacin at 300 mg i.v., with an option to switch to 450 mg orally every 12 h, or moxifloxacin at 400 mg i.v., with an option to switch to 400 mg orally once a day (QD). The mean duration of treatment with delafloxacin was 8.4 (±1.93) days, and the mean duration of treatment with moxifloxacin was 8.5 (±1.97) days. In order to be enrolled, patients had to meet entry criteria and to have radiological evidence as well as ≥2 clinical signs and symptoms of CABP, including cough, production of purulent sputum consistent with a bacterial infection, difficulty breathing (dyspnea), and chest pain due to pneumonia. Additional enrollment criteria were consistent with FDA guidance (11). Efficacy was evaluated through assessments of signs and symptoms of infection 96 (±24) h after the first dose of the study drug (early clinical response [ECR]), with response defined as an improvement in at least 2 of the following symptoms and no worsening of the other symptoms: pleuritic chest pain, frequency or severity of cough, amount and quality of productive sputum, and dyspnea (difficulty breathing). A key efficacy endpoint was the clinical and microbiological assessment at the test of cure (TOC) (5 to 10 days after EOT).

### Analysis sets.

Various subsets of data were used to evaluate the clinical response and the microbiological response. Details of the data sets used for the analysis of the microbiological response are included here. The intent-to-treat (ITT) analysis data set included all patients who signed consent and were randomly assigned to a treatment. The microbiological intent-to-treat (MITT) analysis data set included all patients in the ITT analysis data set who had a baseline bacterial pathogen identified that was known to cause CABP and against which the study drug had antibacterial activity. The MITT population had 2 subgroups, MITT-1 and MITT-2, depending upon the methods of detection of baseline pathogens. MITT-1 consisted of baseline pathogens detected by all methods (i.e., including culture, serology, PCR, and urinary antigen testing), while MITT-2 included baseline pathogens isolated by culture only (blood or any respiratory source, including organisms cultured from oropharyngeal [OP] and nasopharyngeal swabs). The microbiologically evaluable (ME) analysis data set included all subjects in the MITT analysis data set who also met the criteria for the corresponding clinically evaluable (CE) analysis data set. All subjects in the microbiological analysis data sets were analyzed according to their assigned treatment.

### Microbiological outcomes.

By-patient microbiological responses at TOC were determined by consideration of the microbiological response(s) for each baseline pathogen at TOC. By-patient microbiological success was defined as the eradication or presumed eradication of all baseline pathogens. By-patient microbiological responses for patients in the MITT-1 and microbiologically evaluable at test-of-cure (ME-1TOC) populations were based upon by-pathogen microbiological responses of baseline pathogens identified by all test methods. By-patient microbiological responses for patients in the MITT-2 and ME-2TOC populations were based upon by-pathogen microbiological responses of baseline pathogens identified by culture methods.

By-pathogen microbiological responses were based upon follow-up cultures performed at TOC that documented the eradication or persistence of pathogens detected at baseline. When postbaseline culture results were missing, the microbiological response was determined by the clinical outcome assigned by the investigator. Pathogens identified at baseline by a test method other than routine culture of a blood or lower respiratory tract sample (i.e., all atypical pathogens and S. pneumoniae detected by NP swab culture/PCR or UATs) could have only a presumed or indeterminate microbiological response unless persistence (S. pneumoniae only) was demonstrated by traditional culture at EOT or microbiological TOC.

The definitions of documented eradicated, presumed eradicated, and documented persistence were as follows. For documented eradicated, the respiratory and/or blood culture specimen at TOC showed that the pathogen(s) present at enrollment was eradicated, and there was no use of additional antimicrobial therapy for the current infection. For presumed eradicated, no respiratory and/or blood culture specimen was available at TOC with a clinical assessment of success. For documented persistence, the respiratory and/or blood culture specimen collected at TOC was positive for the causative pathogen(s) present at enrollment. Persistence of the baseline pathogen at EOT was carried forward to TOC. For presumed persistence, no respiratory or blood culture specimen was available for a case classified as clinical failure (including failures carried forward to TOC).

### Pathogen detection methods and level of diagnostic certainty.

Pathogens were classified as definitive or probable based on the method of detection (Table 6). If a pathogen was detected or identified from multiple sources and there was at least 1 definitive diagnosis, the pathogen was considered definitive; if all diagnoses were probable, the pathogen was considered probable. Patients with at least 1 definitive diagnosis were considered to have a definitive microbiological diagnosis, and if all diagnoses were probable, the patient was categorized as having a probable microbiological diagnosis. All sputum and endotracheal or transtracheal aspirate (ETA) samples were evaluated by Gram staining to determine specimen quality. All efforts were made to obtain an adequate specimen, defined in this study as having <10 squamous epithelial cells (SECs) and/or >25 polymorphonuclear neutrophils (PMNs) per low-power field. Gram stain quality was used in the evaluation of diagnostic certainty (Table 6).

**TABLE 6:** Pathogen identification and level of microbiological evidence of CABP by detection method^*a*^

Pathogen	Specimen type(s)	Method(s) of detection^*b*^	Criterion(a) for definitive diagnosis	Criterion(a) for probable diagnosis
S. pneumoniae	Sputum and ETA	Culture and Gram stain	Positive culture with Gram staining of <10 SECs and/or >25 PMNs/lpf	Positive culture without Gram staining of <10 SECs and/or >25 PMNs/lpf
	Lavage fluid (BAL and mini-BAL), PSB, pleural fluid, and blood	Culture	Positive culture	
	NP swab	PCR		Positive *lytA* PCR (≥1,000 gene copies/ml)
	NP swab	Culture and PCR	Positive culture with only *lytA* PCR (≥1,000 gene copies/ml)	
	Urine	Urinary antigen test	Positive urinary antigen test	

Other CABP pathogens	Sputum and ETA	Culture and Gram stain	Positive culture with Gram staining of <10 SECs and/or >25 PMNs/lpf	Positive culture without Gram staining of <10 SECs and/or >25 PMNs/lpf
	Lavage fluid (BAL and mini-BAL), PSB, pleural fluid, and blood	Culture	Positive culture	

Mycoplasma pneumoniae	Oropharyngeal swab	Culture	Positive culture	
	Serum	Serology	4-fold increase in titer reaching ≥160	

Legionella pneumophila	Sputum, lavage fluid (BAL and mini-BAL), PSB, and pleural fluid	Culture	Positive culture	
	Urine	Urinary antigen test	Positive urinary antigen test	
	Serum	Serology	4-fold increase in titer reaching ≥128	

Chlamydia pneumoniae	Serum	Serology	4-fold increase to a titer of ≥64	

a Organisms recovered by culture were reviewed on a case-by-case basis by the sponsor prior to database lock to determine eligibility as a CABP pathogen. Subjects who were nasopharyngeal (NP) culture positive with corresponding *lytA* PCR showing <1,000 copies per ml were considered carriers of S. pneumoniae unless S. pneumoniae was detected by another method. Abbreviations: BAL, bronchoalveolar lavage; CABP, community-acquired bacterial pneumonia; ETA, endotracheal or transtracheal aspirate; lpf, low-power field; PMNs, polymorphonuclear neutrophils; PSB, protected specimen brush; SEC, squamous epithelial cells.

b For serology testing, any subject with a 4-fold increase between subsequent visits was considered to have a positive baseline pathogen, even if the 4-fold increase was between 2 postbaseline visits.

In study ML-3341-306, 78/520 (15%) patients in the MITT-1 analysis set had a probable diagnosis. For the microbiological outcome analyses presented here, only the most conservative definitive diagnosis data are presented.

### Microbiology methods.

Causative pathogens were identified by culture and nonculture methods as shown in Table 6. For S. pneumoniae isolates cultured from NP swabs, a concomitant *lytA* PCR value of ≥1,000 gene copies/ml was required for the isolate to be considered a pathogen (13). All isolates underwent susceptibility testing, and a subset of isolates underwent molecular or phenotypic characterization, including whole-genome sequencing for fluoroquinolone resistance mechanisms, PCR for Panton-Valentine leukocidin (PVL) and *mecA* genes (S. aureus [all isolates were PVL negative]) and β-lactamases (*Haemophilus* and *Moraxella* spp.), and serotyping (S. pneumoniae). Details regarding these methods are described below.

### (i) Susceptibility testing.

Isolates were submitted to the central laboratory (Covance Laboratories, Indianapolis, IN, USA) for identification and susceptibility testing according to CLSI guidelines (14). The comparator fluoroquinolone antibiotics included levofloxacin, ciprofloxacin, and moxifloxacin. Nonsusceptibility to these antibiotics was determined using CLSI interpretative criteria (15). For analysis tables prepared using patient outcome and isolate microbiological data, fluoroquinolone susceptibility/nonsusceptibility was based upon levofloxacin and ciprofloxacin data. The designation of S. aureus isolates as MRSA or MSSA was based upon oxacillin susceptibility and cefoxitin disk diffusion results, determined using CLSI interpretative criteria (15). For M. catarrhalis, CLSI document M45 (16) was used. For H. influenzae and *H. parainfluenzae*, MIC interpretations for moxifloxacin, levofloxacin, and azithromycin were derived according to CLSI document M100-S28 (17).

### (ii) Mycoplasma pneumoniae culture.

OP swabs obtained at baseline were forwarded frozen (−60°C) in SP4M transport medium (SP4 media for mycoplasma and ureaplasma isolation) to the University of Alabama (UAB), Birmingham, AL, for M. pneumoniae culture, identification, and antibiotic susceptibility testing. Cultures were performed according to previously described methods (18). Positive mycoplasma broth cultures were subjected to real-time repMp1 (repetitive DNA element in the M. pneumoniae genome) quantitative PCR (qPCR) analysis to differentiate M. pneumoniae from commensal mycoplasma species. Cultures were held for 6 weeks before being reported out as negative.

### (iii) Mycoplasma pneumoniae susceptibility testing.

Antibiotic susceptibility testing for delafloxacin and comparator antibiotics (levofloxacin, moxifloxacin, tetracycline, erythromycin, azithromycin, and clindamycin) was performed using broth microdilution in accordance with CLSI guideline M43-A (19). Microdilution plates were incubated aerobically at 37°C and examined daily for color change in the growth control wells. MIC values were recorded as the lowest concentration of the antimicrobial that inhibits color change in SP4 broth at the time when the growth control well demonstrated a color change from pink to yellow. Assay quality control (QC) was performed each day of antimicrobial susceptibility testing using M. pneumoniae M129 (ATCC 29342). Since there are no delafloxacin M. pneumoniae QC ranges, S. aureus ATCC 29213, E. coli ATCC 25922, and P. aeruginosa ATCC 49247 QC ranges were used according to CLSI document M100-S28 (17). All results were within acceptable QC ranges.

### (iv) Legionella pneumophila culture.

Respiratory samples obtained at baseline were forwarded frozen (−70°C) to the Special Pathogens Laboratory, Pittsburgh, PA, for L. pneumophila culture, identification, antibiotic susceptibility testing, and serotyping. All respiratory specimens were plated on buffered charcoal yeast extract media containing l-cysteine (BCYE), BCYE selective agar with PAC (polymyxin B, anisomycin, and cefamandole; Remel, San Diego, CA, USA) and BCYE selective agar with PAV (polymyxin B, anisomycin, and vancomycin; Remel, San Diego, CA, USA). Some respiratory samples that grew heavy normal flora without *Legionella* species were pretreated with acid (0.2 M KCl-HCl [pH 2.2] acid treatment solution) and recultured. Plates were incubated at 35°C ± 2°C for up to 7 days and examined with the aid of a dissecting microscope. The identification of colonies that resembled those of L. pneumophila was confirmed by using latex agglutination (*Legionella* latex test; Oxoid, Hampshire, UK) and direct immunofluorescence (Monofluo L. pneumophila immunofluorescence assay [IFA] kit; Bio-Rad, Hercules, CA, USA).

### (v) Legionella pneumophila susceptibility testing.

MIC testing was conducted by broth microdilution according to CLSI guidelines for aerobic bacteria (20), using 96-well microtiter plates containing delafloxacin and comparator agents. The bacterial inoculum was prepared from a culture grown overnight on BCYE agar at 35°C ± 2°C in a humidified chamber. The adjusted broth culture was diluted to approximately 0.5 × 10^6^ to 1 × 10^6^ CFU/ml in *Legionella* broth medium (buffered yeast extract broth not supplemented with charcoal) (21). Twofold serial dilutions of antibiotics were prepared in broth (0.05 ml) and added to an equal volume of the inoculum in each well. The final volume per well was 0.1 ml. After incubation at 35°C ± 2°C in a humidified chamber, the MIC was read as the first well showing no visible growth at 48 h. Since there are no delafloxacin L. pneumophila QC ranges, S. aureus ATCC 29213 and E. coli ATCC 25922 QC ranges were used according to CLSI document M100-S28 (17). All results were within acceptable QC ranges.

### (vi) Molecular analysis.

A pathogen was considered fluoroquinolone nonsusceptible if it was nonsusceptible to levofloxacin, ciprofloxacin, or moxifloxacin based on CLSI and EUCAST interpretive criteria. At the time of the study, EUCAST fluoroquinolone breakpoints were lower than CLSI breakpoints; therefore, analyses of fluoroquinolone-resistant pathogens based on EUCAST criteria were chosen for discussion. Whole-genome sequencing was performed using total genomic DNA as the input material for library construction. DNA libraries were prepared using the Nextera XT library construction protocol and index kit (Illumina, San Diego, CA, USA) and sequenced on a MiSeq sequencer (Illumina) using MiSeq reagent kit v3 (600 cycles) with a minimum of 20× coverage. For DNA assembly and data analysis, FASTQ format sequencing files for each sample set were quality assured, error corrected, and assembled independently using the *de novo* assembler SPAdes 3.9.0. A JMI Laboratories-designed software pipeline was applied to the assembled sequences to align them against known plasmid-mediated fluoroquinolone resistance genes (data not shown). *gyrA* and *gyrB* (which encode DNA gyrase) and *parC* and *parE* (which encode topoisomerase IV) sequences were extracted from assembled genomes, and the respective putative protein sequences were screened for mutations in the quinolone resistance-determining regions (QRDRs). Reference sequences for each gene and species were used for comparison with query sequences.

### (vii) Pulsed-field gel electrophoresis.

Isolated genomic DNA of *H. parainfluenzae* was prepared from agarose-embedded cells. Clean extracted DNA was digested with the species-specific restriction enzyme according to the manufacturer’s instructions (New England Biolabs, Ipswich, MA, USA). DNA fragments were resolved on CHEF DR II electrophoresis equipment (Bio-Rad, Hercules, CA, USA) along with the appropriate molecular weight ladders. Gels were stained with ethidium bromide and visualized and documented using the GelDoc XR^+^ system (Bio-Rad).

### (viii) *lytA* PCR assay.

The *lytA* PCR assay is a laboratory-developed test that targets the autolysin gene *lytA*, a single-copy gene that is carried by all pneumococcal strains (22, 23). Sequences of the primers and probe and assay conditions were previously described, with NP swabs used as specimen types (24).

### (ix) Streptococcal and *Legionella* urinary antigens.

Alere BinaxNOW S. pneumoniae and L. pneumophila urinary antigen card tests (Alere, Inc., Scarborough, ME, USA) were performed by Covance Laboratories according to the manufacturer’s directions.

### (x) S. pneumoniae serotyping.

All S. pneumoniae isolates cultured from NP swabs or received from Covance Laboratories were serotyped by the Quellung reaction using Neufeld reagents (Statens Serum Institute, Copenhagen, Denmark) at Emory University. Nontypeable isolates were also tested by latex agglutination and confirmed to be nontypeable using Quellung antisera (results not shown).

### (xi) Serological testing.

Serum samples were collected at baseline (acute samples) and at TOC or follow-up (convalescent samples) and forwarded to the Covance Central Laboratory for serology testing to identify patients infected with atypical pathogens (C. pneumoniae, M. pneumoniae, and L. pneumophila). Serum was tested for anti-C. pneumoniae antibodies using the Focus *Chlamydia* MIF IgG test system (Focus Diagnostics, Cypress, CA, USA). Serum was tested for anti-M. pneumoniae antibodies using MBL Bion M. pneumoniae antigen substrate slides, IgG binding reagent, and reagents for IFAs (MBL, Woburn, MA, USA). Serum was tested for anti-L. pneumophila antibodies using the Zeus IFA L. pneumophila (groups 1 to 6) test system (Zeus Scientific, Branchburg, NJ, USA).

## Supplementary Material

Supplemental file 1
