# Supplementary material for: Efficacy of Delafloxacin versus Moxifloxacin against Bacterial Respiratory Pathogens in Adults with Community-Acquired Bacterial Pneumonia (CABP): Microbiology Results from the Delafloxacin Phase 3 CABP Trial
Source: Antimicrob Agents Chemother. 2020 Feb 21;64(3):e01949-19. doi: 10.1128/AAC.01949-19 (PMC7038307; doi:10.1128/AAC.01949-19)
Supplement: Supplemental file 1 [file AAC.01949-19-s0001.pdf]

Supplementary Appendix

Fold-difference Between Delafloxacin and Moxifloxacin Activity by MIC Against Baseline Pathogens (MITT-2; Delafloxacin and Moxifloxacin Treatment Groups)

**Baseline Target Pathogen**	**MITT-2 Population (Treatment Groups Combined) N = 404**							
	**Delafloxacin**				**Moxifloxacin**			**MIC_{90} Fold-Difference**
	**n**	**MIC Range (µg/mL)**	**MIC_{50} (µg/mL)**	**MIC_{90} (µg/mL)**	**MIC Range (µg/mL)**	**MIC_{50} (µg/mL)**	**MIC_{90} (µg/mL)**	
**Gram-positive organisms**								
Streptococcus pneumoniae	142	0.004–0.03	0.015	0.015	0.06–0.25	0.12	0.25	16
Staphylococcus aureus	57	0.001–0.12	0.002	0.004	0.03–2	0.06	0.12	32
MSSA	55	0.001–0.12	0.002	0.004	0.03–2	0.06	0.12	32
MRSA	2	0.002–0.004	—	—	0.06–0.06	—	—	—
**Gram-negative organisms (fastidious)**								
Haemophilus parainfluenzae	75	0.0005–4	0.008	0.5	0.015–128	0.12	8	16
Haemophilus influenzae	61	0.00025–0.5	0.001	0.002	0.015–16	0.03	0.06	32
Moraxella catarrhalis	12	0.002–0.015	0.004	0.004	0.06–0.5	0.06	0.12	32
**Atypical organisms**								
Mycoplasma pneumoniae	19	0.125–0.5	0.25	0.5	0.125–0.25	0.125	0.25	2
Legionella pneumophila	5	0.00025–0.001	—	—	0.03–0.03	—	—	—

Abbreviations: — = not applicable; MIC_{50} = 50th percentile of MIC values from all pathogens; MIC_{90} = 90th percentile of MIC values from all pathogens; MRSA = methicillin-resistant *S. aureus*; MSSA = methicillin-susceptible *S. aureus*; n = number of pathogens; MIC_{50} and MIC_{90} values were calculated only when 10 or more isolates were available.
